# Editorial: Risk and protective factors in the natural history of autoimmunity

**DOI:** 10.3389/fimmu.2026.1771091

**Published:** 2026-01-16

**Authors:** Christine G. Parks, Esther O. Erdei, Frederick W. Miller

**Affiliations:** 1 Chronic Disease Epidemiology Group, Epidemiology Branch, National Institute of Environmental Health Sciences, National Institutes of Health, Durham, NC, United States; 2 University of New Mexico Health Sciences Center College of Pharmacy, Albuquerque, NM, United States; 3 Environmental Autoimmunity Group, Clinical Research Branch, National Institute of Environmental Health Sciences, National Institutes of Health, Bethesda, MD, United States

**Keywords:** autoimmune diseases, autoimmunity, environmental risk factors, genetic factors, sex differences, triggers and determinants

Autoimmune diseases, the third-most common category after cancer and heart disease, affect at least 5% of the U.S. population (1) and are severe, chronic, and costly to individuals and society. Preclinical or asymptomatic autoimmunity may arise years before diagnosis, occurs in the general population, and appears to be increasing; an example is the rising prevalence of antinuclear antibodies in the U.S. in recent decades (2). However, only some individuals will develop symptoms and pathologies. The articles in this Research Topic focus on risk and protective factors for asymptomatic or preclinical autoimmunity and disease. The relationship between autoimmunity and other diseases, especially cancer and infections, also has important clinical implications. These questions take on greater urgency, given the apparent rise in rates and costs of many autoimmune diseases (3).

## Autoimmunity

Clinical suspicion may lead to autoantibody testing; however, a low predictive probability can result in repeated, costly, and unnecessary testing. Barnado et al. addressed this problem using electronic health records of antinuclear antibody (ANA)-positive individuals, finding a greater likelihood of developing autoimmune diseases among those who were younger, female, with higher-titer ANAs, higher platelet counts, disease-specific autoantibodies, and more billing codes for relevant symptoms. In sum, this model is a useful clinical tool for identifying high-risk ANA-positive patients who should undergo further evaluation, while reassuring lower-risk individuals and reducing unnecessary referrals.

While autoantibodies are known to precede numerous autoimmune diseases, the majority of studies lack longitudinal sampling, and the factors that determine progression or regression are poorly understood. In children at risk of developing type 1 diabetes with disease-specific autoantibodies, Carry et al. found differences in DNA methylation, comparing those who progressed to disease, those who maintained autoantibodies, and those who sero-reverted. The candidate genes were related to diet, glucose levels, and immune and pancreatic beta cells. This suggests that environmental factors may contribute to disease risk. Further studies are needed that include exposure data and biomarkers in the progression of preclinical autoimmunity.

In a cross-sectional analysis of cotinine (a marker of cigarette smoke exposure) and ANA prevalence among a representative population sample of the U.S. population, Dinse et al. observed that, over the study periods (1988-1991, 1999-2004, and 2011-2012), the percentage of individuals with ANA was highest (13.3-19.2%) among nonsmokers but non-trending, lower (11.1-15.5%) for “passive” smokers but steadily increasing, and lowest for active smokers, increasing from 7.4% in 1999–2004 to 13.3% in 2011-2012. These findings imply the presence of unmeasured environmental influences on ANA prevalence.

## Autoimmunity and cancer

In their review of the cancer risk associated with connective tissue disease, Tonutti et al. explored the multiple, complex interrelationships between these entities. The long-recognized increased cancer risk in many rheumatic conditions may develop for various reasons, including loss of immune tolerance due to oncogenesis, proinflammatory immune activation/autoimmunity that may promote oncogenesis, or immunosuppressive therapies that may decrease cancer surveillance. Conversely, autoimmunity may contribute to the removal of constantly generated neoplasms. Incomplete data support all these theories, and further research is needed. In response, Chen highlighted the need for multidisciplinary collaborations that synthesize different diseases and harmonize methods for detecting autoantibodies.

## Sex differences in autoimmunity

Female sex is associated with ANA prevalence and an increased risk of many autoimmune diseases. Investigating a polygenetic risk score for juvenile idiopathic arthritis (JIA), Haftorn et al. examined scores in a population-based study of 238 JIA cases vs. over 73,000 controls. Their investigations into how to best model genetic susceptibilities revealed strong sex differences, suggesting that generalized additive models (GAM) should employ sex stratification, although general linear models can also be applied successfully.

Scofield et al. examined the mechanisms underlying sex differences in immune cells’ Toll-Like Receptor (TLR7) signaling using published studies among subjects with SLE (along with other autoimmune diseases). The authors found that the sex bias among patients was explained by specific gene expressions, while inactivations of the X chromosome were also observed. Examined environmental factors included EBV infections and hormonal, mainly estrogen, effects on B cells, suggesting potential molecular pathways.

## Environmental and genetic risk factors for autoimmune diseases

In their overview, Choi et al. highlighted diverse non-genetic risk and protective factors for systemic autoimmune rheumatic disorders and the complex interactions that may occur prior to disease development. These risk factors include airborne, waterborne, workplace/occupational, social, and behavioral factors, many of which have changed dramatically in recent decades, which may help explain the increase in autoimmunity and disease. Machine learning methods and multiomics have paved the way for a better understanding of these risk factors, and expansions of these and other new technologies could allow for better preventive approaches in the future.

In a study of JIA, Dåstøl et al. explored the role of seafood and dietary contaminants in the context of a polygenic risk score. While they did not find evidence of associations between estimated intakes of environmental contaminants and risk of JIA based on quantiles of fish intake or proxies for potential heavy metal exposure, patients with low genetic predisposition had stronger, significant associations with environmental toxicants, suggestive of environmentally induced JIA.

Some environmental factors may be considered triggers. Concerns have been raised that autoimmunity may develop following vaccine-specific immune activation and inflammatory responses. In their study of myositis patients, Alhassan et al., in the pre-COVID era, found genetic risk and protective factors for developing myositis within 6 months of vaccination. These factors included human leukocyte antigen (HLA) alleles and immunoglobulin (Ig) allotypes. Large-scale studies with greater genotyping and phenotyping are needed to personalize risk assessment and enhance vaccine safety.

Infections are also possible triggers. In a global network of 74 healthcare organizations and nearly 4 million patients, Hileman et al. investigated the incidence of autoimmune diseases up to 1 year after a diagnosed infection. They found an elevated risk of eight autoimmune diseases in patients diagnosed with COVID-19, especially cutaneous vasculitis, polyarteritis nodosa, and hypersensitivity angiitis. A positive ANA was also more likely and predictive of risk following infection. The authors concluded that SARS-CoV-2 may be a potential trigger for some autoimmune diseases, but the risk may diminish over time, as seen in this study following infection with Omicron variants.

## Summary

Taken together, these studies highlight the importance of considering environmental factors and genetic susceptibility in the context of autoimmunity and disease. These contributions suggest the need for well-designed, multidisciplinary studies of asymptomatic autoimmunity, exposome-genome interactions, and relationships with cancer and infections. The external exposome includes a broader range of features than represented here, including heavy metals, other xenobiotics, along with the psychosocial environment and natural disasters (4, 5), all of which warrant focused future research.
